# Fucoidan from *Fucus vesiculosus* alleviates MetALD via promoting HIF-1α ubiquitination to suppress peripheral monocyte infiltration

**DOI:** 10.3389/fphar.2025.1617175

**Published:** 2025-08-15

**Authors:** Lei Li, Jialiang Luo, Di Wang, Yuan Chang, Chaohui Duan, Daming Zuo

**Affiliations:** ^1^ Clinical Laboratory, Sun Yat-sen Memorial Hospital, Sun Yat-sen University, Guangzhou, China; ^2^ Institute of Molecular Immunology, Key Laboratory of Infectious Diseases Research in South China, Ministry of Education, Guangdong Province Key Laboratory of Immune Regulation and Immunotherapy, School of Laboratory Medicine and Biotechnology, Southern Medical University, Guangzhou, China; ^3^ Dermatology Hospital of Southern Medical University, Guangzhou, China; ^4^ Department of Neurology, Nanfang Hospital, Southern Medical University, Guangzhou, China

**Keywords:** fucoidan, infiltrating macrophage, MetALD/ALD, HIF-1α, ubiquitination. Name forward sequence (5'-3') reverse sequence (5'-3') HIF-1α

## Abstract

Metabolic and alcohol-related liver disease (MetALD), characterized by excessive alcohol intake in individuals with metabolic dysfunction, is a growing health burden worldwide. Hepatic macrophages play a pivotal role in MetALD pathogenesis, with pro-inflammatory infiltrating monocytes/macrophages contributing to liver injury. Fucoidan, a sulfated polysaccharide derived from brown algae, is known for its anti-inflammatory properties, yet its intracellular targets remain poorly defined. Here, we identify prolyl hydroxylase domain-containing protein 2 (PHD2) as a novel intracellular binding partner of fucoidan. Using a high-fat diet plus ethanol-induced MetALD mouse model, we demonstrate that fucoidan significantly attenuates hepatocyte injury, steatosis, and peripheral monocyte infiltration in a dose-dependent manner. *In vitro*, fucoidan markedly suppressed ethanol- and LPS-induced THP-1 monocyte migration. Mechanistically, we show that fucoidan binds directly to PHD2, enhancing proteasome-mediated ubiquitination and degradation of HIF-1α, a key transcription factor driving monocyte recruitment and inflammation. Our findings reveal a previously unrecognized mechanism by which fucoidan exerts its anti-inflammatory effects via targeting the PHD2–HIF-1α axis, offering a promising therapeutic strategy for MetALD.

## 1 Introduction

Metabolic and alcohol related liver disease (MetALD) is a major worldwide health issue and its incidence is increasing (Laswi et al., 2022; Rinella et al., 2023). MetALD is the third leading etiologic factor among patients on waiting lists for liver transplantation (Ochoa-Allemant et al., 2025), and the factors that trigger the development of MetALD in these patients remain unknown. Patients with MetALD exhibit distinct clinical characteristics compared to those with metabolic-associated fatty liver disease (MASLD). The characteristics of MetALD are similar to those of alcohol-related liver disease (ALD) (Yoon et al., 2025). In this study, we used a high fat diet combined with alcohol-induced injury in a mouse model to mimic human MetALD. Hepatic macrophages, including Kupffer cells (KCs) and non-KC infiltrating macrophages (IMs), act as sensors of alcohol-induced microbial and metabolic changes, responding by generating signals that regulate all cellular functions in the liver (Ju and Mandrekar, 2015; Szabo, 2015; Wen et al., 2021). Alcohol-induced hepatic inflammation results from a combination of pro-inflammatory IMs infiltrating the liver (Wang et al., 2014) and KC activation (Thurman, 1998). Studies have shown that after 16 weeks of ingestion of a Western diet combined with an ALD diet, KCs are partially derived from monocytes, yet they remain predominantly non-inflammatory. The proportion of IMs in the total number increases from 15% to 50%, with pro-inflammatory and fibrotic features (Ramachandran and Tacke, 2025). Clinical studies have demonstrated that atypical monocytes circulating in the blood of patients with alcoholic hepatitis reduce the recruitment of infiltrating monocytes to the liver, ultimately alleviating alcoholic hepatitis (Rasmussen et al., 2021). Therapies targeting macrophages and interfering with monocyte recruitment through inhibition of chemokine pathways, such as CCR2 or CCL2 (Tacke, 2017), hold great potential in the treatment of liver disease.

Fucoidan, composed of L-fucose and sulphate groups, is a water-soluble acidic heteropolysaccharide enriched in brown algae, exhibiting prominent anti-tumor, anti-thrombosis, anti-oxidative, and anti-inflammatory biological functions. The bioactivities of fucoidans vary depending on their molecular weights and sulfate group contents. A previous study demonstrated that fucoidan (70 kDa, 10% sulfate) upregulated CD206 and IL-10 expression in mouse BMDMs after cytokine stimulation. *In vivo*, fucoidan reversed LPS-induced inflammation (Wang et al., 2023). The immunomodulatory properties of photopolymerized fucoidan were evaluated in human monocytes differentiated into macrophages, where it decreased LPS- and IFN-γ-induced CD86 expression, exhibiting activity similar to IL-10 (Amin et al., 2020). However, the exact function and intrinsic mechanism of fucoidan from *Fucus vesiculosus* in MetALD remain unclear.

HIF-1 is a heterodimeric transcription factor composed of HIF-1α and HIF-1β subunits. Activation of HIF-1α has been reported to be critical for the migration and activation of myeloid cells *in vivo*. It has been shown that HIF-1α is directly related to macrophage migration (Cramer et al., 2003; Palazon et al., 2014). Inhibition of the macrophage HIF-1α-PDK1 axis suppresses systemic inflammation, suggesting a potential therapeutic approach for regulating inflammatory processes (Semba et al., 2016). The absence of HIF-1α in the myeloid lineage results in an almost complete ablation of the inflammatory response in the skin (Cramer et al., 2003). PHD-2 is the hydroxylase most associated with the oxygen-dependent degradation of HIF-1α (Berra et al., 2003; Chen et al., 2017).

This study investigated the role of fucoidan supplementation during the initiation of MetALD. The results showed that fucoidan reduced the infiltration of circulating monocytes into the liver and mitigated the inflammatory response *in vivo*. Furthermore, *in vitro* experiments confirmed that fucoidan physically interacted with PHD2, enhancing the ubiquitination of HIF-1α and subsequently inhibiting the migration of THP-1 cells. Together, these findings uncover a novel mechanism by which fucoidan regulates monocyte infiltration during inflammation. This suggests that fucoidan may serve as a potential therapeutic approach for MetALD and other inflammatory conditions.

## 2 Method and materials

### 2.1 Mice

Wild-type (WT) C57BL/6J mice were acquired from the Animal Institute of Southern Medical University (Guangzhou, China). The mice were housed under specific pathogen-free conditions at a constant temperature (19–23°C) and humidity (55% ± 10%). All animal experiments were approved by the Welfare and Ethical Committee for Experimental Animal Care of Southern Medical University.

Male mice (10–12 weeks old) were chronically fed a high-fat diet and administered binge ethanol to develop a murine model of MetALD. We modified the previous ALD model (Bertola et al., 2013) with a 35% liquid fat diet and alcohol feeding. All mice were fed a liquid control diet (Lieber-DeCarli formulation; Bioserv, Flemington, NJ) for 5 days. The MetALD groups were then fed a liquid diet containing 35% liquid fat diet and 3% v/w ethanol for 10 days, while control mice were pair-fed with the MetALD-fed counterparts for 10 days. On day 11, the MetALD groups received a single oral gavage of ethanol (3 g/kg body weight, 31.25% ethanol), whereas control mice received an isocaloric gavage of dextrin maltose. Fucoidan or phosphate-buffered saline (PBS) was administered via oral gavage. After euthanasia, blood and tissue samples were collected for further analysis.

### 2.2 Antibodies and reagents

Antibodies against HIF-1α (20960-1-AP), PHD2 (19886-1-AP), PDK1 (18262-1-AP) and β-actin (20536-1-AP) were obtained from Proteintech (Rosemont, IL, United States). Anti- Ubiquitin antibody (sc-8017) was purchased from Santa Cruz Biotechnology (Santa Cruz, CA, United States). Anti-Na-K-ATPase (3010S) was purchased from Cell Signaling Technology (Danvers, MA, United States). Percoll (P1644), Tyramine (T90344), Sodium Cyanoborohydride (190020), FITC (1.24546), fucoidan from *F. vesiculosus* (F5631) and Dexamethasone (D4902) were purchased from Sigma-Aldrich (St. Louis, MO, United States). SiRNA targeting HIF-1α (si-HIF-1α) and siRNA targeting PHD2 (si-PHD2) were obtained from RiboBio (Shanghai, China). The fucoidan (F5631) is derived from *F. vesiculosus*, a type of brown algae. The fucoidan is in crude powder form, and it is typically extracted from the brown algae using appropriate purification techniques: Extraction involves treatment of the weed with hydrochloric acid at 70° for one hour at pH 2.0–2.5. The crude fucoidan is isolated by fractional precipitation with alcohol, and purified by treatment with formaldehyde (Black et al., 1953). The monosaccharide composition is not explicitly listed in the certificate, but fucoidan from Fucus vesiculosus typically contains fucose, along with small amounts of galactose, glucose, xylose, mannose, and sulfate groups. The molecular weight of this batch of fucoidan is measured by Multi-angle Laser Light Scattering (MALLS), and the range is between 20,000 and 200,000 Da, with a measured value of 39,700 Da for this specific batch.

### 2.3 Cell culture and stimulation

THP-1 cells (ATCC, Manassas, VA) were cultured in DMEM supplemented with 10% FBS, 100 μg/mL streptomycin, and 100 U/mL penicillin in a humidified 5% CO_2_ atmosphere. THP-1 cells were seeded in a 6-well plate and stimulated with 50 nM EtOH and 100 ng/mL LPS for 24 h (Wu et al., 2023). Fucoidan was added to THP-1 cells 6 h before EtOH and LPS treatment. Dexamethasone (5 uM, Sigma) was used for 1 h before stimulation with EtOH and LPS as positive control (Chen et al., 2024).

### 2.4 Cell apoptosis assay

The effect of fucoidan on cell apoptosis was detected using an Annexin V-FITC/PI apoptosis detection kit (MultiSciences, Hangzhou, China), according to the manufacturer’s instructions. THP-1 cells were seeded into 6-well plates overnight and then cultured in media containing fucoidan. The cells were collected and resuspended in 100 μL of Binding Buffer containing 5 μL Annexin-V and 10 μL PI for 15 min in the dark. Data were acquired on LSRII/Fortessa flow cytometer (BD Biosciences, Heidelberg, Germany) and analyzed with FlowJo software (Tree Star, Ashland, OR, United States).

The effect of EtOH and LPS on cell apoptosis was detected using CCK-8 kit (40203ES60, YEASEN, Shanghai, China), according to the manufacturer’s instructions. THP-1 cells were seeded into 96-well plates overnight and then cultured in media containing different contents of EtOH (0–100 nM) and LPS (0–200 ng/mL). The cells were collected and resuspended in 100 μL of CCK-8 kit in 37°C for 45 min in the dark. Data were acquired on Bio TEK Microplate Reader (Bio Tek Instruments, Inc., United States) and analyzed with FlowJo software (Tree Star, Ashland, OR, United States).

### 2.5 Cell migration assay

THP-1 cells (2 × 10^5^ cells per well) in 100 μL of serum-free medium containing ethanol (EtOH), lipopolysaccharide (LPS), or fucoidan were seeded in the upper chamber. The lower chamber contained the same medium with 15% FBS. After 24 h of incubation, the migrated cells in the lower chamber were collected, counted, and photographed.

### 2.6 Histological and immunohistochemical staining

Paraffin-embedded liver tissue blocks were cut into 5-μm-thick slices and mounted onto poly-L-lysine-coated glass slides. Mouse liver sections were processed for hematoxylin and eosin (H&E) staining and Oil Red O staining according to the standard protocols.

### 2.7 Flow cytometry

The mononuclear cells (MNCs) in the liver were assayed using an LSRII/Fortessa flow cytometer (BD Biosciences, Heidelberg, Germany). Briefly, hepatic MNCs were prepared as previously described (Tang et al., 2018). Liver MNCs or peripheral blood cells were blocked by CD16/32 (14–0161–82, Invitrogen, Waltham, MA, United States) and then stained with Pacific Blue- or FITC-conjugated anti-mouse CD11b antibody (eBioscience, San Diego, CA, United States), PE-conjugated anti-F4/80 antibody (eBioscience) or FITC-conjugated anti-Ly6G antibody (eBioscience). Finally, 7-AAD viability staining solution (eBioscience) was used. FlowJo software (Tree Star, Ashland, OR, United States) was used to analyze the flow cytometric data.

### 2.8 Immunoblotting and immunoprecipitation

RIPA lysis buffer containing protease inhibitors (Beyotime) was used to extract total protein. Protein samples were separated on polyacrylamide gels and transferred onto polyvinylidene fluoride (PVDF) membranes (Millipore, Billerica, MA, United States). Nonspecific binding sites on the membranes were blocked with 5% bovine serum albumin (BSA) for at least 1 h at room temperature. Subsequently, the membranes were incubated with primary antibodies overnight at 4 °C, followed by incubation with horseradish peroxidase-conjugated secondary antibody for 1 h at room temperature. Lastly, the membranes were washed three times, and the target proteins were detected using enhanced chemiluminescence (Thermo Fisher, Carlsbad, CA, United States). ImageJ software (NIH, Bethesda, MD) was used to quantify the densities of protein blots.

The cell lysates were incubated for immunoprecipitation with the indicated antibody and protein A/G PLUS-agarose (sc-2003, Santa Cruz Biotechnology) at 4°C overnight. The beads were washed three times with PBS, resuspended in loading buffer, and boiled for 5 min. Eluted immunoprecipitants were resolved by SDS-PAGE and analyzed for the associated proteins using specific antibodies.

### 2.9 Isolation of RNA and qRT-PCR analysis

Trizol (ET101-01, Transgen, Beijing, China) was used to extract total RNA from liver tissues or cultured cells. Subsequently, RNA was transcribed into cDNA using the reverse transcription kit (TaKaRa, Dalian, China), as instructed by the manufacturer. SYBR Green quantitative RT-PCR was performed to determine the gene expression levels using a 7900HT fast real-time PCR system (Applied Biosystems, San Francisco, CA, United States), according to the protocol provided with SYBR Premix EX Taq (TaKaRa, Dalian, China). The levels of the target gene were normalized to β-actin gene expression. The primer sequences for the genes are presented in Table 1.

**TABLE 1:** Primers used for the ubiquitination real-time PCR in this study.

Name	Forward sequence (5’ – 3’)	Reverse sequence (5’ – 3’)
HIF-1α	ACC​TTC​ATC​GGA​AAC​TCC​AAA​G	ACT​GTT​AGG​CTC​AGG​TGA​ACT
β-actin	GGC​TGT​ATT​CCC​CTC​CAT​CG	CCA​GTT​GGT​AAC​AAT​GCC​ATG​T

### 2.10 Serum biochemical analysis assay and enzyme-linked immunosorbent assay (ELISA)

Serum levels of alanine aminotransferase (ALT), aspartate aminotransferase (AST), triglyceride (TG), total cholesterol (TCH), and hepatic TG levels were measured according to the instructions of commercial assay kits from the manufacturer (Jiancheng Biotech, Nanjing, China).

The commercial ELISA kits for TNF-α (88–7324–22), MCP-1 (88–7503–22), and IL-1β (88–7013–22) were purchased from eBioscience. The levels of cytokines in mouse sera and culture supernatants were detected using the ELISA kits, following the manufacturer’s protocols.

### 2.11 FITC-labeled fucoidan

FITC labeling of fucoidan was performed as previously described (Luo et al., 2022). In brief, 100 mg of fucoidan (F5631, Sigma Aldrich, United States) was dissolved in 3.75 mL of 0.2 M PBS (pH = 8), followed by the addition of tyramine (100 mg) and sodium cyanoborohydride (37.5 mg). After a 96-h incubation at 37°C with intermittent shaking, ethanol (80% v/v) was added to the supernatant, and the precipitate was collected by centrifugation. The precipitate (100 mg) was dissolved in 5 mL of 0.5 M NaHCO_3_, followed by the addition of FITC (12.5 mg). After overnight incubation at room temperature in the dark, ethanol (80% v/v) was added, and the precipitation was collected by centrifugation to obtain the FITC-labeled fucoidan.

### 2.12 Confocal microscopy

Cells were plated on poly-D-lysine-coated glass coverslips and exposed to LPS (100 ng/mL) and alcohol (50 nM) for 12 h, with pretreatment of FITC-FUC for 3 h. Cells were fixed with 4% paraformaldehyde, permeabilized with 0.5% Triton X-100 (Solabio, Beijing, China) in PBS for 8 min, and stained with primary antibodies. The primary antibodies were detected using Alexa Fluor 647-conjugated donkey anti-rabbit IgG, Alexa Fluor 568-conjugated donkey anti-rabbit IgG, or Alexa Fluor 488-conjugated donkey anti-goat IgG. All the images were acquired with a ×20 objective on an Olympus IX81 FV1000 Laser Scanning confocal microscope (Shinjuku, Tokyo, Japan).

### 2.13 Statistical analysis

All results are expressed as mean ± SD. Statistical significance between two groups was evaluated using the Student’s t-test, while comparisons between multiple groups were assessed by two-way analysis of variance (ANOVA), followed by the Student-Newman-Keul’s test. P < 0.05 was considered significant.

## 3 Results

### 3.1 Protective effects of fucoidan from *Fucus vesiculosus* in alcohol-induced murine liver damage

**FIGURE 1:**
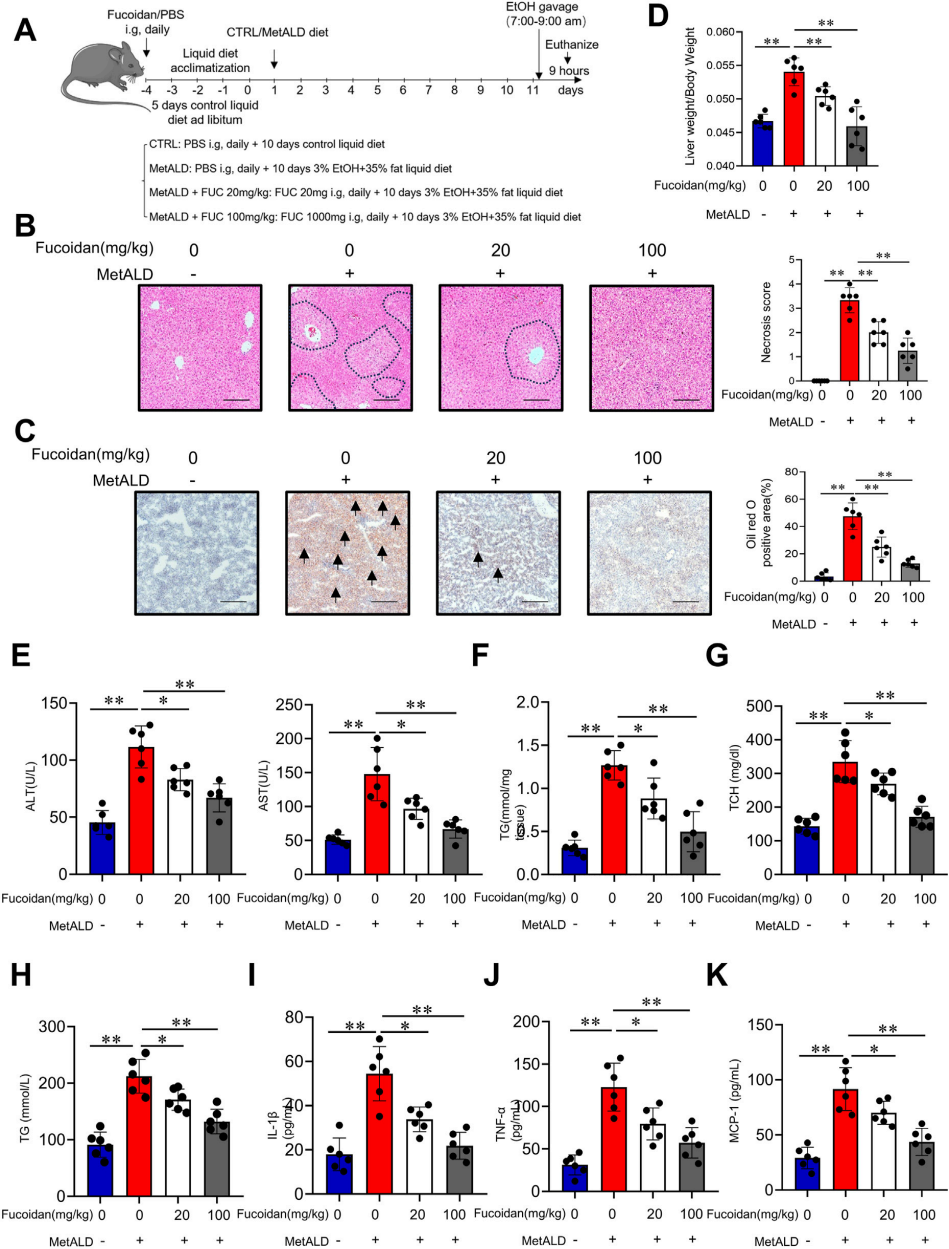
Protective effect of fucoidan from *Fucus vesiculosus* in a mouse model of MetALD. C57/BL6J mice (n = 6 mice/group) were fed a pair-fed control diet or a 35% fat plus 3% ethanol diet with or without fucoidan for 2 weeks. **(A)** Overview of the MetALD mice model procedure. **(B)** Histological analysis of liver sections was performed using H&E staining. Circles indicate the sites of injury. Scale bars = 100 µm. **(C)** Lipid accumulation was assessed by Oil Red O staining of liver sections. Arrows indicate lipid droplet deposition. Scale bars = 100 µm. **(D)** Body and liver weights were measured, and the liver/body weight ratio was calculated. **(E)** Serum ALT and AST activities were assessed. **(F)** Hepatic TG content was determined. **(G,H)** Serum TCH **(G)** and TG **(H)** levels were measured. **(I–K)** Serum levels of IL-1β **(I)**, TNF-α **(J)** and MCP-1 **(K)** were quantified by ELISA. ns: not significant, *p < 0.05, **p < 0.01, determined by unpaired Student’s t-test. Data are representative of three independent experiments with similar results.

To explore the potential role of fucoidan from *F. vesiculosus* in MetALD, mice were chronically fed a high-fat chow diet plus binge ethanol feeding to establish a MetALD murine model, along with fucoidan administration via intragastric gavage (20 mg/kg, 100 mg/kg) (Lim et al., 2015; Zhao et al., 2021). Our previous experiments have also demonstrated that these concentrations do not impair liver function in mice (Luo et al., 2022). Chronic binge ethanol feeding (Figure 1A) induced hepatocyte damage and hepatic steatosis, along with an increased liver-to-body weight ratio. Notably, fucoidan significantly attenuated these effects in a dose-dependent manner (Figures 1B–D). Moreover, fucoidan supplementation led to a marked reduction in serum alanine aminotransferase (ALT) and aspartate transaminase (AST) levels (Figures 1D,E). Hepatic triglycerides (TG), serum total cholesterol (TCH), and triglycerides (TG) were also significantly decreased in fucoidan-treated mice, with the 100 mg/kg fucoidan showing superior efficacy (Figures 1F–H). The serum levels of IL-1β, TNF-α and MCP-1 were elevated in the MetALD group mice, while they decreased with fucoidan supplementation (Figures 1I–K). Together, these findings suggest that fucoidan has the potential to protect against MetALD injury in mice.

### 3.2 Macrophage infiltration contributes to liver injury in liver damage upon alcohol challenge

**FIGURE 2:**
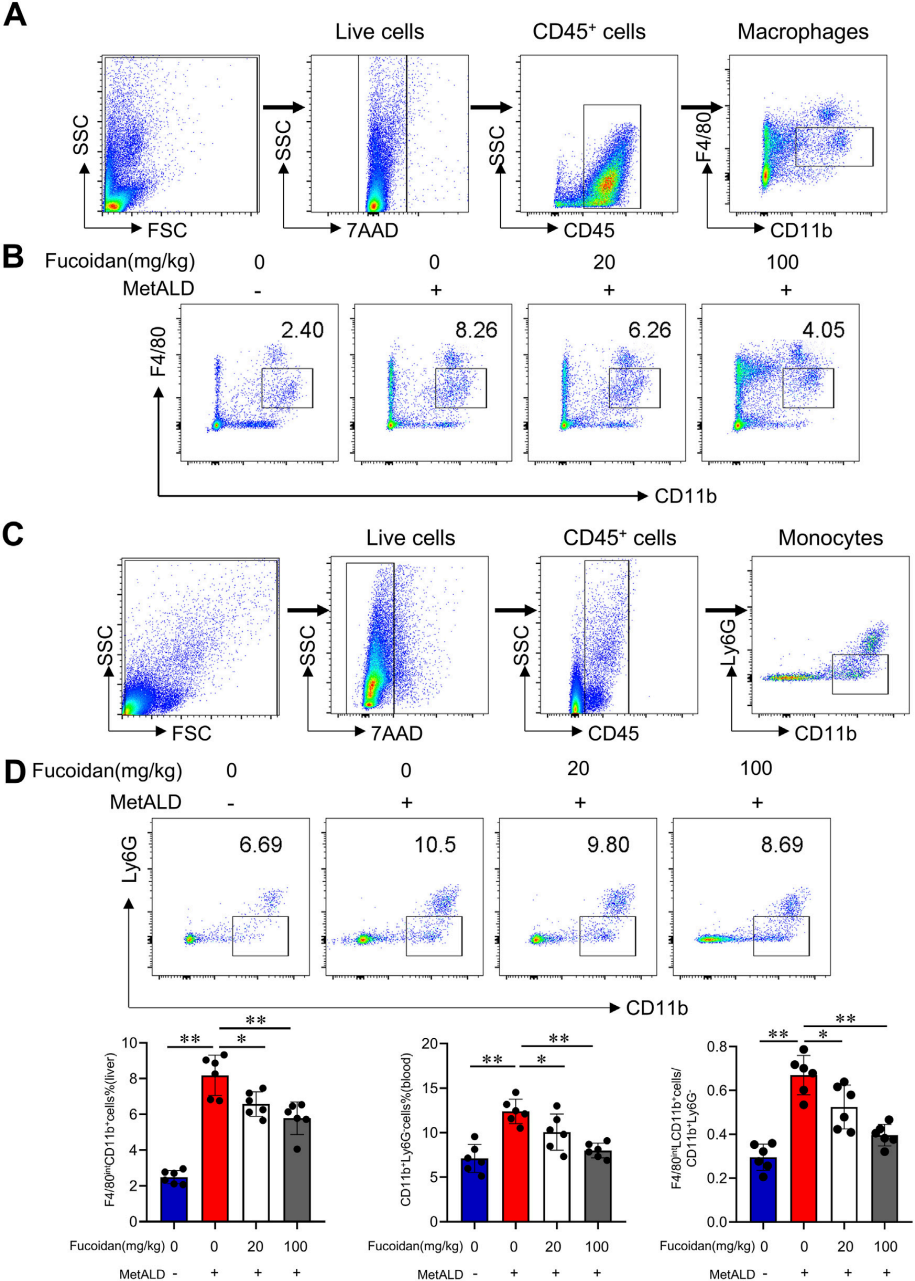
Macrophage infiltration is responsible for the protective effects against liver damage following alcohol challenge. C57/BL6J mice (n = 6 mice/group) were fed a pair-fed control diet or the MetALD diet with or without fucoidan for 2 weeks. **(A)** Gating strategy of macrophage. **(B)** Liver macrophages infiltration in MetALD mice was assessed by staining with anti-CD11b and anti-F4/80 antibodies, followed by FACS analysis. **(C)** Gating strategy of monocyte. **(D)** Peripheral blood monocytes were evaluated by staining with anti-CD11b and anti-Ly6G antibodies, followed by flow cytometry analysis. ns: not significant, *p < 0.05, **p < 0.01, determined by unpaired Student’s t-test. Data are representative of three independent experiments with similar results.

In alcoholic liver disease, the infiltration of macrophages into the liver during chronic injury is closely associated with the progression of liver inflammation in both mice and humans (Duffield et al., 2005; Karlmark et al., 2009; Karlmark et al., 2010; Zimmermann et al., 2010). We investigated the effects of fucoidan on macrophage infiltration in MetALD mice models. Flow cytometry analysis (Figures 2A,B) identified infiltrating macrophages (CD11b^+^F4/80^int^). Chronic binge ethanol feeding increased infiltrating macrophages, while fucoidan remarkably reduced the frequency of infiltrating macrophages in the livers of mice. Consistently, the frequency of monocytes (CD11b^+^Ly6G^−^) was lower in fucoidan-treated mice compared to the MetALD group in blood (Figures 2C,D), indicating that fucoidan decreased the infiltration of monocytes into the liver during MetALD progression.

### 3.3 Fucoidan from *Fucus vesiculosus* suppresses the migration of monocytes

**FIGURE 3:**
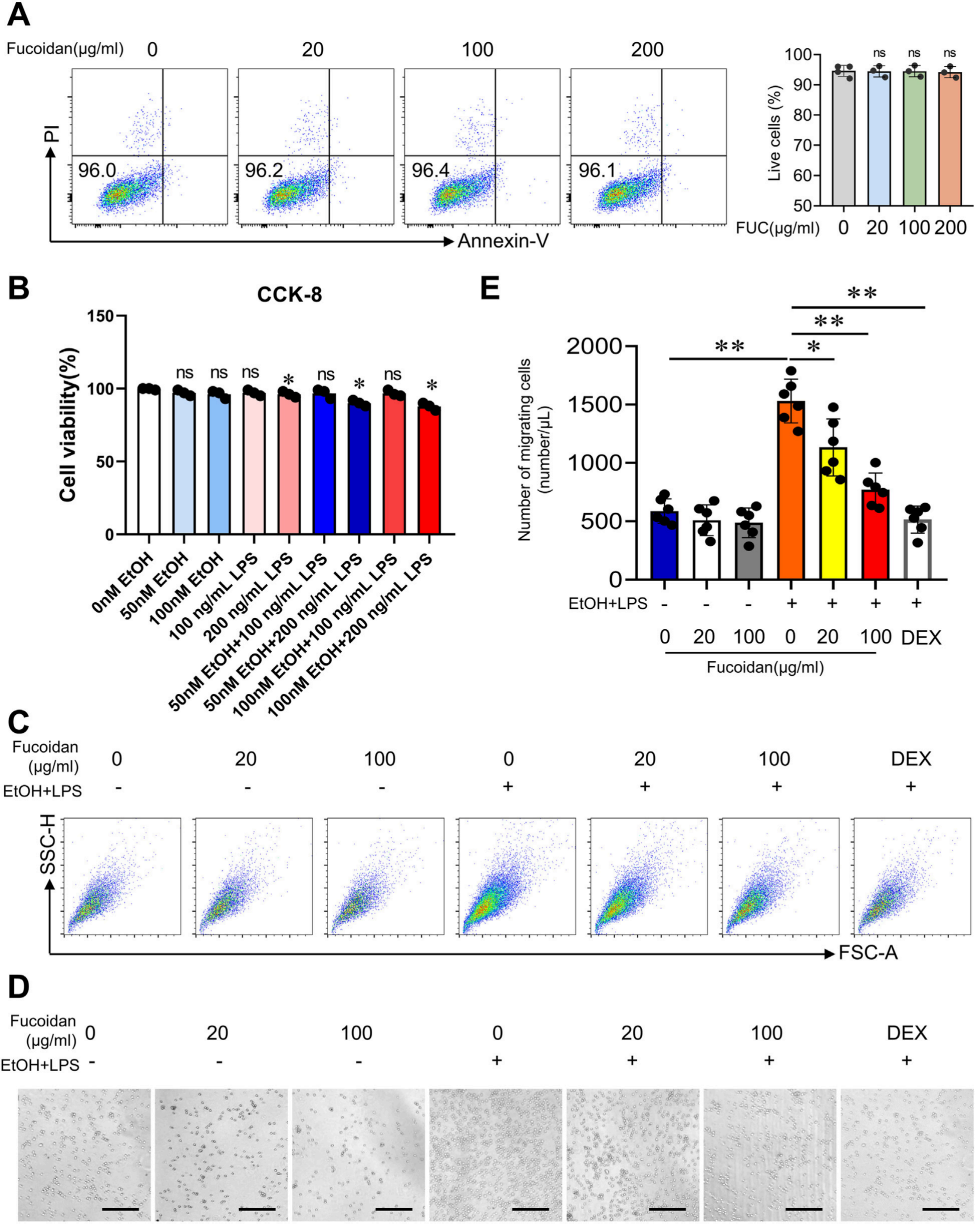
Fucoidan from *Fucus vesiculosus* suppresses monocyte migration. THP-1 cells were pretreated with fucoidan for 6 h before exposure to EtOH and LPS for 24 h. **(A)** THP-1 cells were co-incubated with fucoidan for 24 h. Apoptosis of THP-1 cells was analyzed by FACS. **(B)** THP-1 cells were co-incubated with different concentrations of EtOH and LPS for 24 h. Apoptosis of THP-1 cells was analyzed by CCK-8. Cell migration was assessed by transwell motility assay after the THP-1 cells with or without fucoidan were treated with EtOH and LPS for 24 h. Dexamethasone (4 uM, Sigma) was used for 1 h before stimulation with EtOH and LPS as positive control. **(C)** The number of migrated cells was detected from 1/500 volume of the bottom chamber using flow cytometry. **(D)** Image of migrated cells in the bottom chamber. **(E)** The number of migrated cells was quantified. Scale bars = 100 μm. ns: not significant, *p < 0.05, **p < 0.01, determined by unpaired Student’s t-test. Data are representative of three independent experiments with similar results.

Inflammatory monocytes play a key role in promoting inflammation during alcohol-related liver disease (Tacke, 2012). Inhibition of hepatic monocyte infiltration is a promising therapeutic approach for MetALD. To explore the pharmacological effects of fucoidan on monocyte migration, we conducted *in vitro* experiments using THP-1 cells. First, we tested the effect of fucoidan on cell apoptosis by flow cytometry. Fucoidan treatment did not increase the apoptosis rate in THP-1 cells at doses of 20, 100 or 200 μg/mL (Figure 3A). Then, the effect of EtOH and LPS on cell apoptosis by CCK-8 was tested. We selected 50 nM of EtOH and 100 ng/mL of LPS as safe concentrations to stimulate THP-1 cells. (Figure 3B). Given the disrupted intestinal barrier and increased LPS levels in the liver microenvironment during MetALD (Wu et al., 2023), we hypothesized that EtOH may promote cell migration via activation of chloride channels (Wei et al., 2015), and that lipopolysaccharide (LPS)-induces migration in RAW264.7 cells and bone marrow-derived macrophages (BMDMs) *in vitro* (Gao et al., 2021). We stimulated THP-1 cells with EtOH and LPS, dexamethasone (4 uM, Sigma) was used for 1 h before stimulation with EtOH and LPS as positive control. Transwell assays revealed that fucoidan dose-dependently inhibited the migration of THP-1 cells under EtOH and LPS treatment (Figure 3C). Images and cell counts both confirmed fewer migrated THP-1 cells in the presence of fucoidan compared to the EtOH and LPS group (Figures 3D,E). Overall, these results suggest that fucoidan inhibits monocyte migration induced by EtOH and LPS.

### 3.4 Fucoidan from *Fucus vesiculosus* suppresses the migration of monocytes via the HIF-1α pathway

**FIGURE 4:**
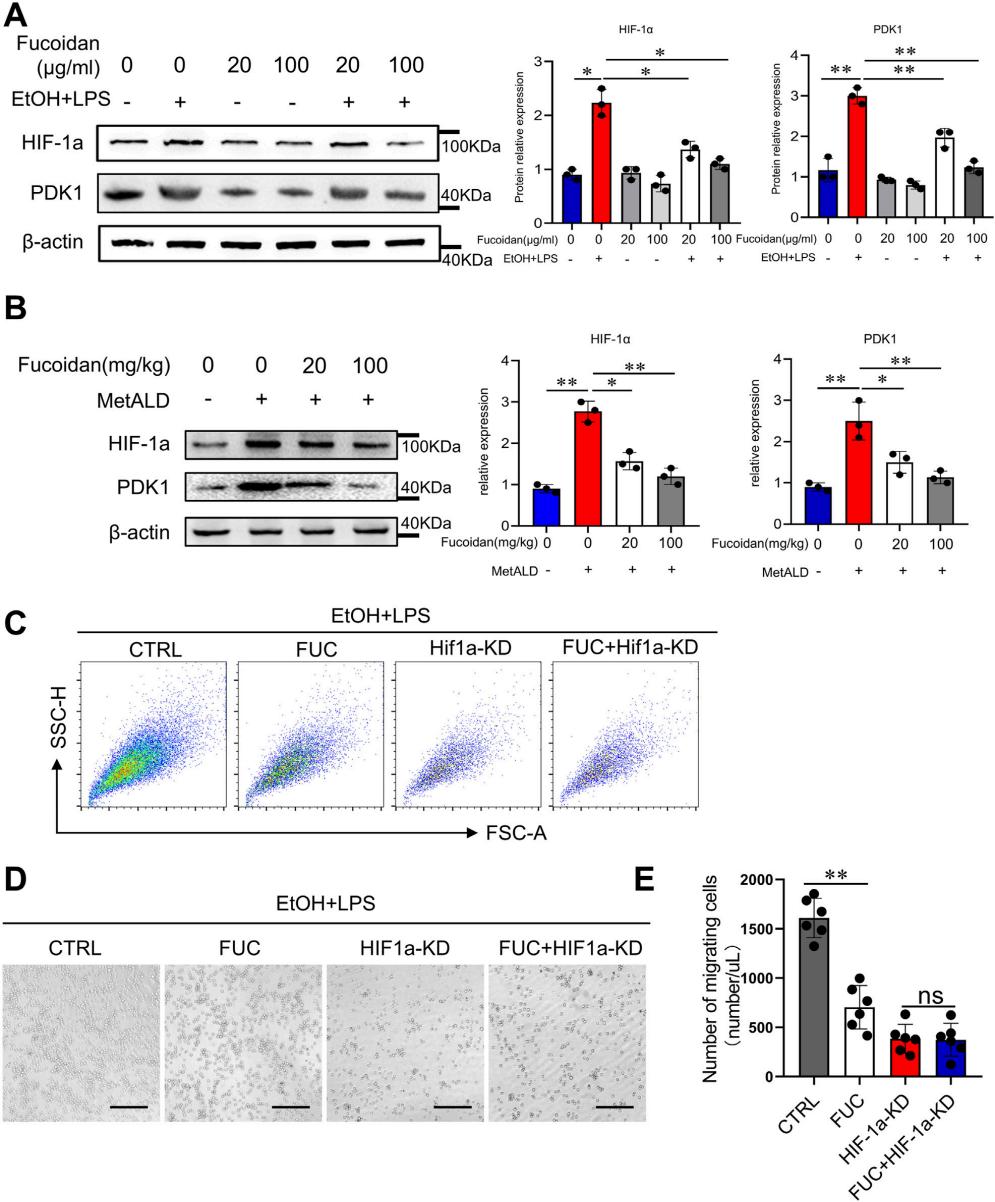
Fucoidan from *Fucus vesiculosus* suppresses monocyte migration via the HIF-1α pathway. **(A)** THP-1 cells were exposed to EtOH and LPS for 24 h with or without pretreatment with fucoidan for 6 h. The protein levels of HIF-1α and PDK1 were evaluated by Western blotting. **(B)** Mice (n = 3 mice/group) were fed a pair-fed control diet or the MetALD diet with/without fucoidan for 2 weeks. The liver MNCs protein levels of HIF-1α and PDK1 were evaluated by Western blotting. **(C–E)** Cell migration of THP-1 cells was assessed by transwell assay following treatment with fucoidan and EtOH and LPS, and HIF-1α knockdown. **(C)** The number of migrated cells was detected from 1/500 volume of bottom chamber using flow cytometry. **(D)** Image of migrated cells was acquired. Cell migration was assessed by transwell motility assay. **(E)** Number of migrated cells was quantified. Scale bars = 100 μm. ns: not significant, *p < 0.05, **p < 0.01, determined by unpaired Student’s t-test. Data are representative of three independent experiments with similar results.

Previous studies have shown that HIF-1α is associated with cell migration and invasion (Gao et al., 2020). Glycolytic reprogramming promotes macrophage migration via the HIF-1α-PDK1 axis (Semba et al., 2016). Therefore, we investigated whether fucoidan regulates HIF-1α to inhibit monocyte migration. The results showed that EtOH and LPS stimulation significantly increased HIF-1α expression, while fucoidan decreased *HIF-1α* expression in THP-1 cells in a dose-dependent manner (Figure 4A). Fucoidan also inhibited HIF-1α expression in mononuclear cells (MNCs) isolated from the livers of MetALD mice (Figure 4B). To further confirm that fucoidan regulates HIF-1α, we applied HIF-1α siRNA to interfere with HIF-1α expression. The results of transwell assays (Figure 4C), images of migrated cells (Figure 4D), and the number of cells that migrated to the lower chamber (Figure 4E) confirmed that knockdown of HIF-1α significantly decreased the migration of THP-1 cells and abolished the differences between the groups with or without fucoidan. These findings suggest that the HIF-1α-PDK1 signaling pathway could be a potential target of fucoidan in regulating cell migration. Taken together, these results suggest that fucoidan reduces monocyte migration via the HIF-1α signaling pathway.

### 3.5 Fucoidan from *Fucus vesiculosus* downregulates HIF-1α expression via the ubiquitination pathway

**FIGURE 5:**
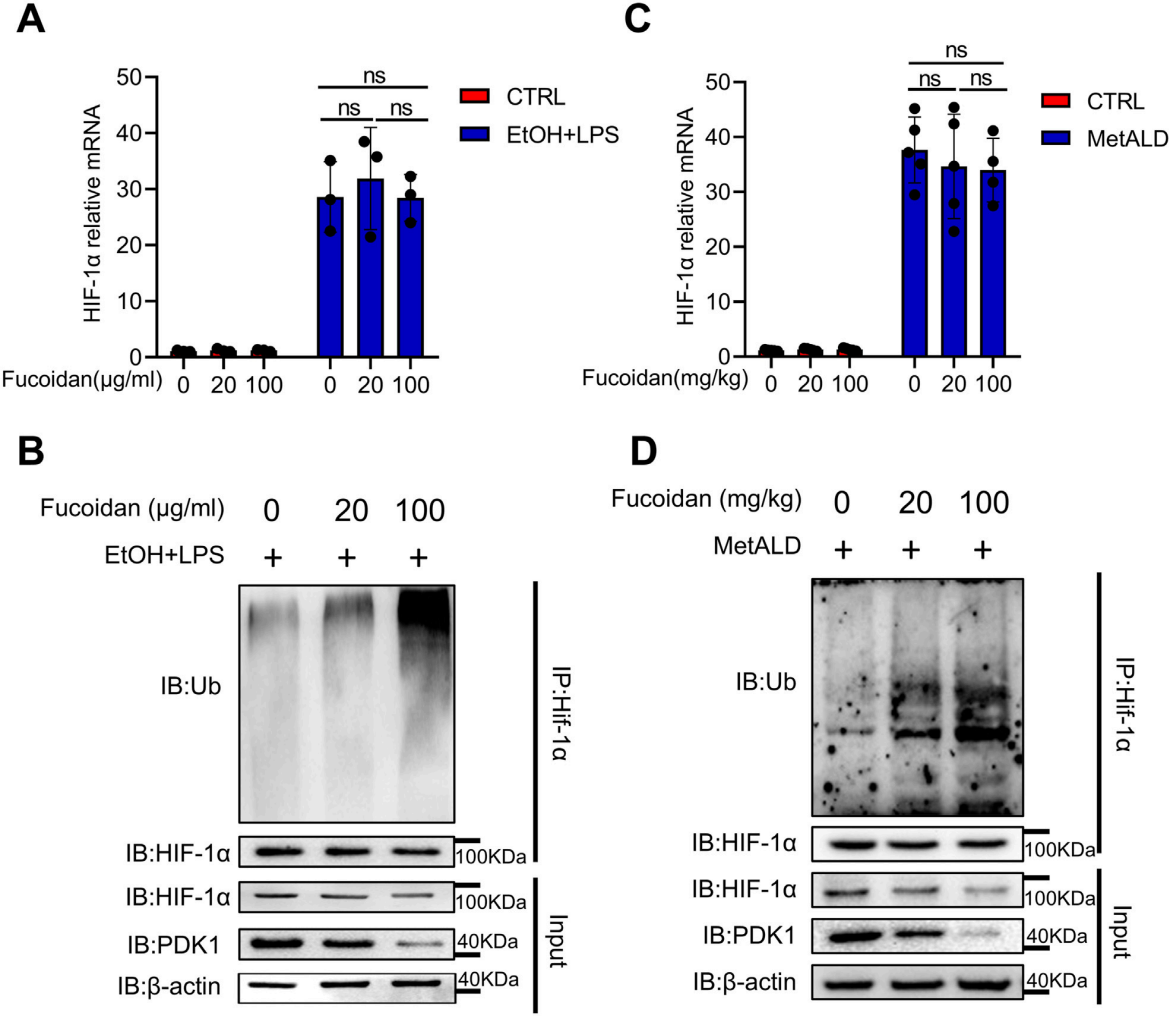
Fucoidan from *Fucus vesiculosus* downregulates the HIF-1α expression via the ubiquitination pathway. THP-1 cells were treated with EtOH and LPS for 24 h with or without fucoidan pretreated for 6 h. **(A)** The mRNA levels of *HIF-1α* was tested by qRT-PCR. **(B)** The protein levels of HIF-1α ubiquitination was evaluated by Western blotting. C57/BL6J mice (n = 5 mice/group) were fed a pair-fed control diet or the MetALD diet with or without fucoidan for 2 weeks. **(C)** The mRNA level of *HIF-1α* was tested by qRT-PCR in MNCs of the liver. **(D)** The protein level of HIF-1α ubiquitination was evaluated by Western blotting in hepatic MNCs. ns: not significant, *p < 0.05, **p < 0.01, determined by unpaired Student’s t-test. Data are representative of three independent experiments with similar results.

An increasing number of proteins and small molecules have been identified to regulate HIF-1α ubiquitination activity by modulating the physical or functional interaction of PHD2, VHL, or HSP90 with HIF-1α (Semenza, 2007). In EtOH and LPS stimulated THP-1 cells treated with or without fucoidan, the mRNA level of HIF-1α showed no significant differences (Figure 5A). Then we focused on the post-translational modifications of HIF-1α. We observed that fucoidan decreased the protein expression of HIF-1α, accompanied by an upregulation of HIF-1α ubiquitination under EtOH and LPS stimulation (Figure 5B). Furthermore, the mRNA level (Figure 5C) and ubiquitination of HIF-1α (Figure 5D) in MetALD liver MNCs treated with fucoidan were confirmed. These results indicate that fucoidan downregulates HIF-1α expression via the ubiquitination pathway, suggesting that fucoidan promotes the ubiquitination of HIF-1α to decrease the activity of the HIF-1α pathway.

### 3.6 Fucoidan from *Fucus vesiculosus* affects the ubiquitination of HIF-1α via PHD2

**FIGURE 6:**
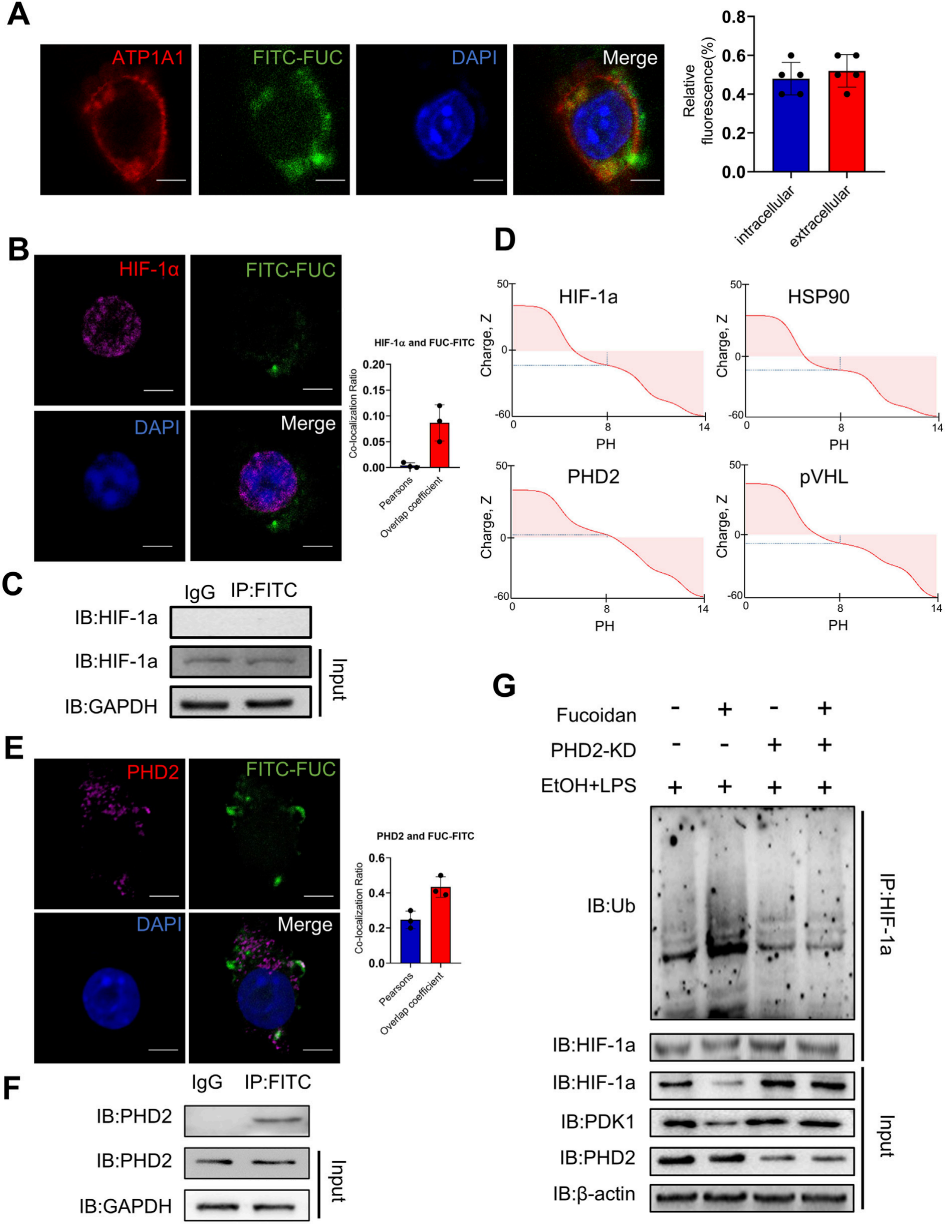
Fucoidan from *Fucus vesiculosus* affects the ubiquitination of HIF-1α via PHD2. THP-1 cells were treated with EtOH and LPS for 12 h with or without fucoidan pretreated for 3 h. **(A)** The co-localization of fucoidan (green) and ATP1A1 (red), a membrane marker, in THP-1 cells treated with EtOH and LPS was determined by confocal microscopy. **(B)** The co-localization of fucoidan (green) and HIF-1α (purple) in THP-1 cells treated with EtOH and LPS was determined by confocal microscopy. **(C)** The THP-1 cells were treated with EtOH and LPS with or without fucoidan. The association of fucoidan with HIF-1α in THP-1 cells treated with EtOH and LPS was determined by immunoprecipitation. **(D)** The isoelectric points of HIF-1α, HSP90, PHD2, and pVHL were predicted using the online tool (https://www.protpi.ch/). **(E)** The co-localization of fucoidan (green) and PHD2 (red) in THP-1 cells treated with EtOH and LPS was determined by confocal microscopy. **(F)** The THP-1 cells were treated with EtOH and LPS with or without fucoidan. The association of fucoidan with PHD2 in THP-1 cells treated with EtOH and LPS was determined by immunoprecipitation. **(G)** The association of HIF-1α and PHD2 with or without fucoidan was determined by immunoprecipitation. Scale bars = 20 µm. Data are representative of three independent experiments with similar results.

Next, we sought to investigate how fucoidan affects the ubiquitination level of HIF-1α. Firstly, we needed to determine whether fucoidan could traverse the cell membrane. In this study, fucoidan was labeled with fluorescein isothiocyanate (*FITC*). Immunofluorescence results showed that FITC-labeled fucoidan (FITC-FUC) was partly transported into THP-1 cells (Figure 6A), which was supported by previous reports indicating that fucoidan immunostaining in liver tissue appeared in an elliptical or club-shaped pattern in non-parenchymal cells in sinusoids but not in parenchymal cells (Nagamine et al., 2014). Then we investigated potential protein interactions with fucoidan. Initially, we tested whether fucoidan could directly bind to HIF-1α. The results (Figures 6B,C) suggested that fucoidan did not bind directly to HIF-1α. HIF-1α ubiquitination is mediated by several proteins, including the von Hippel-Lindau tumor suppressor gene product (pVHL), prolyl hydroxylase (PHD2), and heat shock protein-90 (HSP90). As reported, fucoidan is negatively charged, which enables it to bind positively charged proteins (Figure 6B). Subsequently, we analyzed the isoelectric points of HIF-1α, pVHL, PHD2, and HSP90. As shown in Figure 6B, only PHD2 was positively charged. Consistently, co-localization of fucoidan with PHD2 was observed in THP-1 cells (Figure 6D). Furthermore, co-immunoprecipitation results showed that FITC-fucoidan could physically interact with PHD2 (Figures 6E,F). To further confirm the role of PHD2 in fucoidan’s action, we examined the expression and ubiquitination levels of HIF-1α after treatment with siPHD2. The results showed no significant differences in HIF-1α expression or ubiquitination between the presence and absence of fucoidan following PHD2 knockdown (Figure 6G). These results indicated that fucoidan may traverse the membrane and interact with PHD2, leading to an increase in HIF-1α ubiquitination and a decrease in HIF-1α protein levels. In summary, these findings suggest that fucoidan reduces macrophage infiltration by interacting with PHD2, thereby enhancing the ubiquitination of HIF-1α and inhibiting the HIF-1α pathway.

## 4 Discussion

MetALD is a newly recognized subtype of MASLD induced by excessive alcohol intake. Research on MASLD has provided valuable insights into the role of hepatic macrophages in mediating metabolic injury, inflammation, and fibrosis (Peiseler et al., 2022). Key findings regarding macrophage heterogeneity in MASLD include phenotypic changes in the KC subpopulation (i.e., resident hepatic phagocytes), recruitment of monocyte-derived macrophages to the injured region, and the specific phenotypes of macrophage recruitment to these regions, such as lipid-associated macrophagesor scar-associated macrophages (Hammerich and Tacke, 2023). However, applying the findings to alcohol-related liver disease has proven to be difficult. In this study, we focused on MetALD and employed a high-fat diet combined with alcohol feeding as a model, which is more relevant to the pathogenesis of human MetALD compared to the Western diet plus alcohol regimen (Sasaki et al., 2025). To further investigate alcohol-related mechanisms, we used an alcohol plus LPS treatment protocol in our *in vitro* models.

Inhibition of hepatic monocyte infiltration is a promising therapeutic approach for alcohol related liver disease (Ju and Mandrekar, 2015). Hepatic macrophages are functionally diverse in alcohol-associated liver disease, with KCs and infiltrating monocytes/macrophages each representing approximately 50% of the bone marrow pool. Five major KC clusters express genes involved in receptor-mediated endocytosis and lipid metabolism, with the majority predicted to be non-inflammatory and anti-fibrotic. In contrast, infiltrating monocyte/macrophage clusters are pro-inflammatory and profibrotic (Sasaki et al., 2025). Targeting specific macrophage subpopulations may offer novel strategies for treating liver failure and fibrosis in alcohol liver disease. Fucoidan, a polysaccharide enriched in sulfate groups and primarily found in brown algae, has demonstrated potential therapeutic benefits against various diseases. Previous studies have shown that fucoidan suppresses carbohydrate and lipid metabolism, inflammation, tumor proliferation, and metastasis, establishing it as a promising candidate for liver protection (Zhao et al., 2021). In this study, we demonstrate that fucoidan inhibits the migration of peripheral monocytes into liver macrophages, protecting against liver injury through suppression of the HIF-1α signaling pathway.

Fucoidans represent a class of polysaccharides composed mainly of l-fucose and sulfate ester groups (Li et al., 2008). The sulfate content was not of a crucial importance for the complement activation, but they highlighted the positive influence of fucose residues in the polysaccharide structure for the activation of the alternative pathway of the complement (Menshova et al., 2016), the sulfate content may influence the receptor binding and the following NO production in the anti-inflammatory response (Hou et al., 2020). There are high-molecular-weight fucoidan (HMWF) with MW = 100 ± 4 kDa and low-molecular-weight fucoidan (LMWF) with MW < 30 kDa, HMWF enhanced the severity of arthritis, the inflammatory responses in the joint cartilage, and the levels of collagen-specific antibodies, whereas LMWF reduced arthritis through the suppression of Th1-mediated immune reactions (Park et al., 2010).

Fucoidan has been reported to inhibit the growth and migration of bladder cancer cells (Cho et al., 2014). For example, Sung et al. showed that fucoidan treatment altered the structure of F-actin in filopodial protrusions, and transwell assays confirmed that fucoidan from *Sargassum hemiphyllum* inhibited cell migration (Sung et al., 2022). Our findings are consistent with these reports, as fucoidan from *F. vesiculosus* inhibited THP-1 cell migration under alcohol and LPS exposure via the HIF-1α signaling pathway. This is in line with previous studies suggesting that the antiangiogenic activity of low molecular weight fucoidan (LMWF) in bladder cancer is associated with suppressing HIF-1/VEGF- signaling pathway (Chen et al., 2015).

Although fucoidan has shown promising therapeutic effects, its long-term use could potentially lead to side effects such as immune system suppression or other adverse reactions. While there is limited clinical data available regarding the pharmacokinetics of fucoidan, previous studies have indicated that fucoidan has a relatively low bioavailability when administered orally (Pozharitskaya et al., 2018). Although fucoidan is widely recognized for its safety in applications, it is important to consider that its natural source—brown seaweed—may accumulate toxic elements such as arsenic and cadmium depending on environmental conditions (Matos et al., 2024). While purification processes typically remove most of these contaminants, rigorous quality control and adherence to safety regulations are necessary to ensure the safety of fucoidan products. Future work should focus not only on functional activity but also on ensuring the absence of hazardous substances in the final formulation. Additionally, about its half-life *in vivo*, an ELISA assay showed the concentration detected (median) was 4.002 and 12.989 mg/L when 3 g of 10% or 75% pure fucoidan was ingested orally over a period of 12 days, respectively (Irhimeh et al., 2005). Large molecular weight and solubility may affect its absorption and distribution, potentially limiting its therapeutic efficacy through conventional routes of administration. The biological activity of fucoidan is strongly influenced by its structural characteristics, including the monosaccharide composition, degree of sulfation, and molecular weight. In addition, impurities co-extracted during the isolation process, such as phlorotannins, may also contribute to or interfere with the observed bioactivity. Phlorotannins are polyphenolic compounds derived from brown algae that have been reported to exert antioxidant and anti-inflammatory effects (Catarino et al., 2018), potentially confounding the attribution of biological activity solely to fucoidan. In our study, we used a commercial fucoidan product with a reported purity of ≥95%, suggesting limited levels of non-polysaccharide impurities. However, since detailed compositional analysis was not performed, we acknowledge that trace amounts of phlorotannins or other polyphenolic compounds may still be present and could influence the observed effects. Future studies should include comprehensive chemical profiling (e.g., HPLC, NMR, or LC-MS) to distinguish the individual contributions of fucoidan and co-extracted compounds such as phlorotannins.

To balance the clinical application prospects with the potential risks, we suggest that future studies should focus on the long-term safety of fucoidan, especially with regard to its immune-modulating effects. Moreover, pharmacokinetic studies, including the half-life and biological availability of fucoidan, are crucial to guide its clinical usage and optimize its dosing regimen. The biochemical heterogeneity of fucoidan arising from species variation, geographical origin, and collection timing introduces substantial variability in its bioactivity. Several recent studies have systematically profiled fucoidans from Arctic brown algae and demonstrated that both environmental stressors and reproductive cycles contribute to changes in polysaccharide architecture (Obluchinskaya et al., 2022; Obluchinskaya et al., 2023). These compositional shifts may impact their pharmacological potential, particularly in immune modulation. Thus, while our study highlights the therapeutic benefit of fucoidan, a more detailed compositional analysis will be essential to elucidate structure-function relationships and ensure consistency in future applications.

The HIF-1α signaling pathway plays a crucial role in alcohol-induced liver disease. Binge alcohol consumption promotes acute liver injury in both mice and humans through a CYP2E1-HIF-1α-dependent pathway (Yun et al., 2014). Our study demonstrates that fucoidan reduces inflammation and lipid accumulation in alcohol-related liver disease by modulating the HIF-1α signaling pathway. HIFs are heterodimeric basic helix-loop-helix transcription factors composed of an oxygen-sensitive α-subunit and a constitutively expressed β-subunit (Semenza, 2012). Under normal oxygen conditions, proline residues within the oxygen-dependent domain of HIF-1α are hydroxylated by PHD2 within minutes. This modification targets HIF-1α for ubiquitination and subsequent proteasomal degradation. In hypoxic conditions, the activity of PHDs is inhibited, leading to the stabilization of HIF-1α and an increase in its transcriptional activity. The suppression of PHD2 plays a central role in elevating HIF-1α levels (Berra et al., 2003; Luo et al., 2018). Thus, PHDs regulate HIF-1α stability through oxygen-dependent hydroxylation, which is crucial for its degradation under normoxic conditions. In this study, we show that fucoidan downregulates HIF-1α protein levels by promoting its ubiquitination via PHD2. Previous research has suggested that fucoidan may act as a therapeutic agent in breast cancer through a ubiquitin-dependent degradation pathway affecting the TGFR/Smad/Snail, Slug, Twist and epithelial-mesenchymal transition axes (Hsu et al., 2013). These findings support our results that fucoidan enhances the ubiquitination of certain proteins. However, further investigation is needed to determine the specific domains and key side chains of PHD2 that bind to fucoidan.

Immunostaining of fucoidan in mouse livers has previously revealed its presence in non-parenchymal cells in the sinusoids but not in parenchymal cells (Nagamine et al., 2014). In our study, we labeled fucoidan with FITC (Bai et al., 2020; Luo et al., 2022), and confocal microscopy confirmed that FITC-labeled fucoidan was able to enter monocytes. The negative charge of fucoidan, due to the presence of sulfate groups at the C-2 and C-4 positions, and occasionally in C-3 positions, allows it to form complexes with positively charged molecules (Citkowska et al., 2019). Our findings indicate that fucoidan binds to PHD2, thereby increasing the ubiquitination of HIF-1α. Given that PHD2 is positively charged, this interaction helps explain the mechanism by which fucoidan regulates HIF-1α. While previous reports have focused on the binding of fucoidan to surface receptors, the targets of intracellular fucoidan warrant further investigation.

In summary, our study reveals a critical biological function of fucoidan in reducing the inflammatory response to alcohol exposure, offering a novel and promising strategy for the clinical treatment of MetALD.

## Data Availability

The raw data supporting the conclusions of this article will be made available by the authors, without undue reservation.
